# Hepatitis B Reactivation in a HBsAg-Negative, HBcAb-Positive Patient Receiving Fludarabine for the Treatment of Chronic Lymphocytic Leukaemia

**DOI:** 10.1155/2011/258791

**Published:** 2011-06-15

**Authors:** Federica Toscanini, Pasqualina De Leo, Giuseppe Calcagno, Federica Malfatti, Alessandro Grasso, Marco Anselmo

**Affiliations:** ^1^Department of Infectious Diseases, San Paolo Hospital, Via Genova 20, 17100 Savona, Italy; ^2^Department of Internal Medicine and Gastroenterology, San Paolo Hospital, 17100 Savona, Italy

## Abstract

Hepatitis B virus (HBV) reactivation is an increasingly recognized cause of morbidity and mortality in patients undergoing chemotherapy. In haematology, the risk of reactivation of B hepatitis among HBsAg-positive patients has been documented; therefore, use of lamivudine prophylaxis is recommended before starting chemotherapy. Differently, for HBsAg-negative patients with markers of previous HBV infection (i.e., presence of isolated anti-HBc positivity) (anticore patients) management strategies are not univocal. We describe a rare case of HBV reactivation in an anticore patient after fludarabine therapy for chronic lymphocytic leukaemia. The patient fully recovered after a 6-month course of lamivudine with persistent HBV-DNA clearance and loss of HBsAg. The most important feature of this case is that fludarabine alone infrequently determines HBV reactivation, especially in anticore patients. Therefore, we suggest that patients candidates to receive fludarabine therapy should be considered for lamivudine prophylaxis, not only if HBsAg-positive, but even if anticore-positive only.

## 1. Introduction

Chemotherapy for patients suffering from haematological malignancies usually determines immunosuppression. This condition may expose patients to a variety of opportunistic infections. Among these latter, Hepatitis B virus (HBV) reactivation is an increasingly recognized cause of morbidity and mortality in such patients [1].

In haematology, the risk of reactivation of B hepatitis among HBsAg-positive patients appears to be greater than in other settings of oncology [2], and it has been well documented; therefore, use of lamivudine prophylaxis is widely recommended before starting chemotherapy [2–4]. Differently, for HBsAg-negative patients with markers of previous HBV infection (i.e., presence of isolated anti-HBc positivity) (anticore patients) management strategies are not univocal [3, 4]. Anticore patients present a lower risk of HBV reactivation than HBsAg-positive subjects; nevertheless, such risk can vary depending on the kind of chemotherapeutic agents employed.

Fludarabine, a purine analogue used as a single agent or combined with other drugs in the treatment of various haematological malignancies, is very effective in the treatment of chronic lymphocytic leukaemia (CLL) and indolent non-Hodgkin lymphoma (NHL) [5, 6]. Fludarabine determines a profound and prolonged immunosuppression with a relevant decrease of CD4^+^ and CD8^+^ lymphocytes, which predispose to opportunistic infections [7, 8]. This condition may potentially promote HBV replication in HBsAg-positive subjects. Subsequently, after the end of chemotherapy, it would be the immune reconstitution that causes an immune-mediated destruction of HBV-infected liver cells.

## 2. Case Presentation

A 46-year-old man presented at the Emergency Room of our hospital in May 2009 with vomiting, fatigue, and minimal jaundice. Blood tests showed increased serum alanine transaminase (ALT) at 2,635 UI/mL (range: 0–35 UI/mL), aspartate transaminase (AST) at 1,357 UI/mL (range: 0–35 UI/mL), and total bilirubin at 2.5 mg/dL (range: 0–1.2 mg/dL); for the suspect of acute hepatitis, he was hospitalised at the Infectious Diseases Department of our Hospital. Further serological tests showed the following: Hepatitis C virus (HCV)-Ab and HCV-RNA negative, Human Immunodeficiency virus (HIV)-Ab negative, Hepatitis A virus (HAV)-Ab total positive and IgM negative, HBsAg, HBeAg and HBcAb-total: positive, HBsAb, HbcAb-IgM (below cut off limit 0.8 IU/mL; Architect, Abbott), HBeAb: negative, HBV-DNA 7 × 10^6^ UI/mL (cut off limit 12 UI/mL).

In 2007, the patient had been diagnosed CLL, then he had been followed up and treated at an Haematology Department at another institution in a nearby town for this disease. The patient had received fludarabine only for 6 months between October 2008 and March 2009 with disease remission. Careful review of clinical charts from the other hospital and discussion with the haematologists who had treated him concluded that no HBV screening (no marker, not even HBsAg) had been performed before chemotherapy, and therefore, no anti-HBV prophylaxis was administered. In addition, no haemoderivatives were administered. The patient had been a blood donor at our hospital from 2003 to 2007; he had always been HBsAg negative. HBV-DNA was performed only in pooled samples, but pools containing his blood were always negative for it. An old test positive for HBcAb (2002) was eventually detected in our hospital data bank.

After diagnosing HBV reactivation, lamivudine treatment (100 mg daily) was started. The patient received a full 6-month course of lamivudine with rapid and persistent HBV-DNA clearance, loss of HBsAg and HBeAg, and complete seroconversion with appearance of HBsAb and HBeAb (Table 1).

## 3. Discussion

The described case showed a rare HBV reactivation after the end of a successful 6-month course of fludarabine therapy for CLL.

Most cases of HBV reactivation correlated to fludarabine treatment (during or after such treatment) reported so far have been observed in HBsAg-positive patients [9–13]. This is one of the very few reported cases of HBV reactivation in an anticore patient [8, 9]. Furthermore, most reported reactivation cases, both in HBsAg-positive and anticore patients, had received chemotherapy that included other immunosuppressive agents in addition to fludarabine (most often rituximab) [10–12]. Only extremely rarely HBV reactivation was observed in HBsAg-negative/HBcAb-positive (anticore patients) who received fludarabine only as this patient was [9].

Interestingly, as it had already been reported in other cases, viral reactivation in this patient was observed at the time of expected immunological reconstitution [9]. Actually, more than 50% of hepatitis flare-ups due to HBV reactivation occur in the window between the end of chemotherapy and the complete recovery of immunocompetence [14]. In fact, it has been suggested that after widespread viral infection of hepatocytes during the immunosuppression phase, the rebound in T-cell function and number may lead to massive hepatocyte destruction [15]. This may mean that before chemotherapy, this patient probably had an extremely very low level of circulating HBV-DNA (≤12 copies/mL), which was not detectable by the standard diagnostic tests. Alternatively, he could have harbored variable amounts of HBV-DNA exclusively in the liver, and the virus began to massively replicate upon the breakdown of immunosuppression [15, 16].

As recently suggested also by others, we propose that lamivudine prophylaxis should be recommended (or at least considered) to prevent HBV reactivation in candidates to receive fludarabine therapy, not only for HBsAg-positive patients, but also for those who are HBsAg-negative/HBcAb-positive (anticore patients) [1]. Such prophylaxis should probably be extended after the end of fludarabine treatment to include the immunological reconstitution phase (at least 3 to 6 extra months).

In addition, it must be stressed once more that patients who are candidates for immunosuppressive treatments including drugs associated to hepatitis B-reactivation should be screened not only for HBsAg (and this case was not even tested for this), but also for HBcAb and HBsAb; otherwise, it is not even possible to plan for any prophylaxis. In fact, if this patient had been tested, positivity for anti-HBc would have been probably detected. Then, HBV DNA should have been tested, and, if positive, antiviral therapy started, if negative, close monitoring of ALT and HBV DNA should have been done [3, 4].

In conclusion, this paper suggests that patients HBsAg-negative/HBcAb-positive (anticore patients) constitute a subset at risk of hepatitis due to viral reactivation after fludarabine treatment even when used as single chemotherapeutic agent.

## Figures and Tables

**Table 1:** Serological HBV markers and HBV-DNA at diagnosis, during lamivudine treatment (Months 1, 3, and 6) and at 6-month followup (Month 12) after the end of lamivudine treatment.

	Onset	Month 1	Month 3	Month 6	Month 12
HBsAg	+	+	+	−	−
HBsAb	−	−	+	+	−
HBcAb-total	+	+	+	+	+
HBcAb-IgM	−	−	−	−	−
HBeAg	+	+	−	−	−
HBeAb	−	−	−	+	+
HBV-DNA (UI/mL)	7.06 × 10^6^	5,96 × 10^3^	1.4 × 10^2^	<12	<12

Legend: HBsAg: Hepatitis B surface antigen; HBsAb: Hepatitis B surface antibodies; HBcAb-total: Hepatitis B core antibodies (total); HBcAb-IgM: Hepatitis B core antibodies (IgM only); HBeAg: Hepatitis B e antigen; HBeAb: Hepatitis B e antibodies.
